# Does prior use of antiplatelet therapy modify the effect of dual antiplatelet therapy in transient ischaemic attack/minor ischaemic stroke: A systematic review and meta‐analysis

**DOI:** 10.1111/ene.15433

**Published:** 2022-06-20

**Authors:** Aoibhin Clarke, Catriona Reddin, Robert Murphy, Martin J. O'Donnell

**Affiliations:** ^1^ HRB—Clinical Research Facility National University of Ireland Galway Galway Ireland; ^2^ Galway University Hospital Galway Ireland; ^3^ Wellcome Trust—HRB, Irish Clinical Academic Training Dublin Ireland

**Keywords:** aspirin, dual antiplatelet therapy, stroke, transient ischaemic attack

## Abstract

**Background and purpose:**

The purpose was to determine whether prior use of antiplatelet therapy modifies the effect of dual antiplatelet therapy in patients with acute minor ischaemic stroke or transient ischaemic attack.

**Methods:**

A systematic review and meta‐analysis of randomized controlled trials was performed comparing dual antiplatelet therapy to aspirin that reported subgroup analysis by prior antiplatelet use, adhering to the Cochrane Collaboration Guidelines. A fixed‐effects meta‐analysis was used to estimate a pooled treatment effect overall in subgroups with prior aspirin therapy and without prior aspirin therapy. Difference in treatment effect was assessed by testing *p* for interaction. The primary outcome measure was recurrent vascular events.

**Results:**

Three eligible randomized controlled trials were identified, including 4831 participants with pre‐existing antiplatelet use and 16,236 participants without pre‐existing aspirin use. Recurrent vascular events occurred in 7.2% (95% confidence interval [CI] 4.3–10) of those without pre‐existing aspirin use versus 7.3% (95% CI 4.1–10) of those receiving prior aspirin therapy. Effect of dual antiplatelet therapy on the primary outcome measure was consistent in participants with no prior aspirin use (odds ratio 0.75, 95% CI 0.66–0.84) compared to those taking aspirin before randomization (odds ratio 0.79, 95% CI 0.63–0.998) (*p* interaction = 0.66). The number needed to treat in the aspirin‐naïve group was 55 (95% CI 37‐107) compared to 66 (95% CI 32 to –746) in those on prior aspirin therapy.

**Conclusions:**

It was found that the effectiveness of dual antiplatelet therapy in patients with minor ischaemic stroke or high risk transient ischaemic attack does not significantly differ in patients with prior aspirin exposure; therefore there should be no influence on the decision to use dual antiplatelet therapy.

## Graphical abstract

We performed a systematic review and meta‐analysis of randomised controlled trials comparing dual antiplatelet therapy to aspirin that reported subgroup analysis by prior antiplatelet use. Three eligible randomized controlled trials were identified, including 4831 participants with pre‐existing antiplatelet use and 16,236 participants without pre‐existing aspirin use. We found no evidence to support the contention that prior antiplatelet use modifies the effectiveness of dual antiplatelet therapy in patients with minor ischaemic stroke or high risk transient ischaemic attack, and should not influence clinical decision‐making.

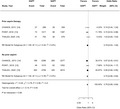

## INTRODUCTION

Dual antiplatelet therapy is recommended for acute management of non‐cardioembolic minor ischaemic stroke and high risk transient ischaemic attack (TIA) within 24 h and at least within 7 days from symptom onset, on the basis of clinical trials and meta‐analyses that have demonstrated a lower rate of subsequent stroke compared with single antiplatelet therapy [1, 2]. A number of clinical factors have been shown to influence effectiveness, including time from symptom onset to initiation of dual antiplatelet therapy [3]. In clinical practice, 26.9% of patients presenting with TIA and ischaemic stroke are already receiving an antiplatelet agent [4]. It is uncertain whether prior use of antiplatelet therapy modifies the efficacy and safety of dual antiplatelet therapy in these patients and therefore whether it should be engaged in clinical decision‐making.

The aim of this meta‐analysis was to determine whether prior use of antiplatelet therapy modifies the effect of dual antiplatelet therapy in patients with acute minor ischaemic stroke or TIA.

## METHODS

A systematic review and meta‐analysis was performed adhering to the Cochrane Collaboration Guidelines and our findings are reported according to the Preferred Reporting Items for Systematic Reviews and Meta‐Analyses (PRISMA) guidelines (Table S1) [5, 6]. The meta‐analysis was registered with the International Prospective Register of Systematic Reviews (PROSPERO identifier CRD42022301292).

### Data sources and search strategy

To reduce research waste data were extracted from a recent meta‐analysis of dual versus single antiplatelet therapy in acute minor ischaemic stroke or TIA [2, 7]. It was considered of sufficiently high quality to avoid the need to repeat it. Our search was limited to dates not included in this review (July 2020 onwards). The systematic review by Bhatia et al. provided a comprehensive analysis comparing the outcomes of early initiation of short‐term dual antiplatelet therapy versus aspirin alone in patients with acute stroke or TIA [2]. The PubMed and Embase databases were systematically searched from July 2020 to 27 December 2021. The search terms included are detailed in Appendix S1 (Methods S1). Following the removal of duplicates, titles and abstracts were screened by two reviewers (AC and CR) using the Rayann web application (Figure S1) [8]. Full texts of the remaining articles were independently assessed for eligibility based on predetermined criteria by two reviewers (AC and CR). Disagreements were resolved by consensus; where a resolution was not reached by discussion, a consensus was reached through a third reviewer (MOD).

### Eligibility criteria

Studies were considered eligible if they (1) included patients with a diagnosis of minor ischaemic stroke or TIA, (2) compared dual antiplatelet therapy to single antiplatelet therapy, (3) reported subsequent stroke/vascular events in patients with and without prior antiplatelet therapy and (4) were randomized controlled trials.

### Data extraction/measurements

Data were extracted independently by two authors (AC and CR) using a standardized pre‐determined data collection form. Data were compared for inconsistencies and merged into a final dataset.

### Outcomes

The primary outcome measure was recurrent vascular events, including all stroke, myocardial infarction or vascular death. Outcome measures differed slightly between trials and are outlined in Table 1.

**TABLE 1:** Study designs and treatment protocols of the included studies

Trial	No. of participants (prior aspirin)	No. of participants (no prior aspirin)	Study population	Outcome	Intervention group	Control group	Follow‐up duration
CHANCE, 2013 [15]	587^a^	4573^a^	Minor stroke (NIHSS ≤3) or TIA with ABCD2 ≥4 within 24 h	Composite of ischaemic stroke, haemorrhagic stroke, myocardial infarction or vascular death	Aspirin and clopidogrel	Aspirin	90 days
POINT, 2018 [14]	2814	2067	Minor stroke (NIHSS ≤3) or TIA with ABCD2 ≥4 within 24 h	Composite of ischaemic stroke, myocardial infarction or death from ischaemic vascular causes	Aspirin and clopidogrel	Aspirin	90 days
THALES, 2020 [16]	1433	9583	Mild–moderate acute non‐cardioembolic ischaemic stroke (NIHSS ≤5), or TIA with ABCD2 ≥6, or symptomatic intracranial or extracranial arterial stenosis within 24 h	Composite of all stroke or death within 30 days	Aspirin and ticagrelor	Aspirin	30 days

Abbreviations: NIHSS, National Institutes of Health Stroke Scale; TIA, transient ischaemic attack.

^a^ Extracted from CHANCE, Figure S5.

### Data synthesis and analysis

A descriptive analysis of trials and baseline characteristics of participants is reported in Table 1. The odds ratio (OR) and 95% confidence interval (CI) for each outcome of interest were calculated from individual studies. Weighted pooled treatment effects were calculated individually for prior aspirin use and no prior aspirin use using restricted maximum likelihood estimation to fit a fixed‐effects meta‐analysis model. Our objective was to determine the difference in treatment effect of dual antiplatelet therapy between populations on aspirin prior to the ischaemic event and those not on aspirin. Difference in treatment effect was statistically tested for by testing a *p* for interaction between subgroups of participants with prior aspirin use and without prior aspirin use. *p* for interaction <0.1 was considered evidence of statistical heterogeneity [9]. Summary estimates were calculated for subgroups with prior aspirin use and without prior aspirin use. Statistical analysis was performed using the Metafor package on R Statistical Software (version 3.6.2) [10].

### Risk of bias assessment

The Cochrane risk of bias tool for randomized trials (RoB 2) was used to assess methodological quality of eligible trials [11]. Risk of bias assessments were performed independently by reviewers (AC and CR) and disagreements were resolved by a third reviewer (MOD). Studies were deemed at high risk of bias overall if one or more domains were rated as high, or if multiple domains were judged to have ‘some concerns in a way that substantially lowers confidence in the result’ [11]. Risk of bias summary tables were generated (Figure S2).

## RESULTS

Three trials were eligible for inclusion with a mean follow‐up duration of 70 days, including 4831 participants with pre‐existing antiplatelet use and 16,236 participants without pre‐existing antiplatelet use. The POINT trial enrolled the highest proportion of participants with prior aspirin therapy at 57.6% of participants, compared to 13.0% of participants in THALES and 11.5% in CHANCE. No studies were deemed to be at high risk of bias (Figure S2).

**FIGURE 1:**
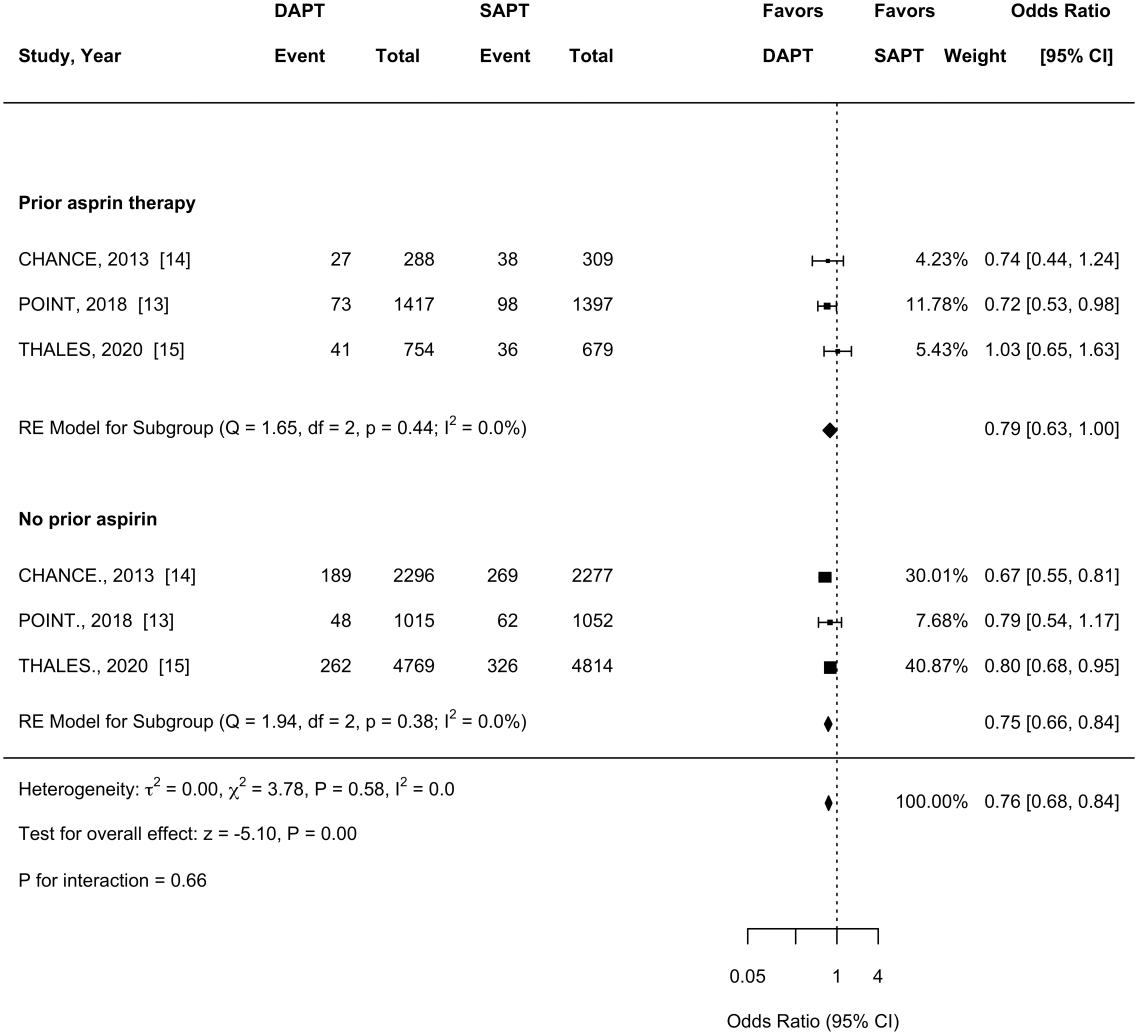
Forest plot demonstrating the association of DAPT and recurrent vascular events. The squares and bars represent the mean values and 95% confidence intervals of the effect sizes, whilst the area of the squares reflects the weight of the studies. The combined effects appear as diamonds and the vertical dashed line represents the line of no effect. CI, confidence interval; DAPT, dual antiplatelet therapy; OR, odds ratio; SAPT, single antiplatelet therapy. (Random effect estimates: prior aspirin therapy OR 0.79, 95% CI 0.63–0.99; no prior aspirin therapy OR 0.75, 95% CI 0.65–0.86)

Recurrent vascular events occurred in 7.3% (95% CI 4.1–10) of those with prior aspirin therapy versus 7.2% (95% CI 4.3–10) of those without pre‐existing aspirin use. In a population without prior aspirin use, dual antiplatelet therapy compared to single antiplatelet therapy was associated with a significant reduction in recurrent vascular events (OR 0.75, 95% CI 0.66–0.84). Similarly, in a population with prior aspirin use, dual antiplatelet therapy compared to single antiplatelet therapy was associated with a significant reduction in recurrent vascular events (OR 0.79, 95% CI 0.63–0.998). There was no evidence of a statistically significant difference between populations (*p* interaction = 0.66) (Figure 1). The number needed to treat in those without prior aspirin use was 55 (95% CI 37–107) compared to 66 (95% CI 32 to ‐746) for those with prior aspirin use.

## DISCUSSION

In this systematic review and meta‐analysis, which included three trials with 21,067 participants, no evidence was found of statistically significant differences in the association of dual antiplatelet therapy with recurrent vascular events between patients with and without prior antiplatelet therapy.

Over a quarter of patients who present with an acute stroke or TIA are prescribed one or more antiplatelet agents prior to the event; however, the relative efficacy of dual antiplatelet therapy compared to single antiplatelet therapy in this population has not been evaluated in prior meta‐analyses [4]. There is considerable variability in antiplatelet prescribing patterns for those who present with an ischaemic stroke whilst on aspirin therapy [12]. Our findings extend those of Anadani et al. which reported dual antiplatelet therapy was associated with similar risk reduction of ischaemic stroke regardless of premorbid antiplatelet use in a post hoc analysis of the POINT trial [13]. Current evidence supports the use of dual antiplatelet therapy over single antiplatelet therapy in the setting of high risk TIA or mild–moderate ischaemic stroke. Four randomized trials show a reduced risk of subsequent stroke, major adverse cardiovascular events and recurrent ischaemic events with dual antiplatelet therapy compared to aspirin therapy [14, 15, 16, 17]. The results of these trials have supported recommendations for early treatment with dual antiplatelet therapy in the standard care of patients with minor stroke or TIA [2, 3]. Our review supports the use of dual antiplatelet therapy in patients with minor stroke or TIA regardless of prior antiplatelet use.

### Limitations of our study

Our meta‐analysis has a number of limitations. It included a small number of trials. These studies enrolled patients with minor strokes or high risk TIAs, which may limit generalizability to those with moderate to severe strokes or lower risk TIAs.

There were a number of differences between trials, including variations in follow‐up, categorization of previous aspirin therapy and primary outcome measures, as detailed below. In contrast to POINT and THALES, the subgroup analyses in CHANCE specified ‘aspirin taken within 24 h’ rather than any previous aspirin therapy which may limit the results of this study. In CHANCE, the primary outcome was new ischaemic or haemorrhagic stroke event at 90 days. As the secondary trial outcome of a composite of ischaemic stroke, haemorrhagic stroke, myocardial infarction or vascular death was more similar to the primary outcomes of the other included trials, this was used for the purpose of our analysis.

## CONCLUSIONS

This meta‐analysis adds to evidence that the association of dual antiplatelet therapy with recurrent vascular events does not differ significantly due to pre‐treatment with aspirin and should be considered in those with minor ischaemic stroke or high risk TIA irrespective of prior aspirin treatment.

## AUTHOR CONTRIBUTIONS

**Aoibhin Clarke:** Conceptualization (lead); data curation (equal); formal analysis (equal); investigation (equal); methodology (equal); project administration (equal); resources (equal); validation (equal); visualization (equal); writing – original draft (equal); writing – review and editing (equal). **Catriona Reddin:** Data curation (equal); formal analysis (equal); investigation (equal); methodology (equal); software (equal); validation (equal); visualization (equal); writing – original draft (equal); writing – review and editing (equal). **Robert Murphy:** Formal analysis (supporting); investigation (equal); methodology (equal); software (equal); validation (equal); visualization (equal); writing – original draft (equal); writing – review and editing (equal). **Martin O'Donnell:** Conceptualization (equal); formal analysis (equal); investigation (equal); methodology (equal); supervision (equal); writing – original draft (equal); writing – review and editing (equal).

## CONFLICT OF INTEREST

Ethics approval and informed consent were not required for this systematic review. The authors have no conflicts of interest to disclose.

## Supporting information


Appendix S1

## Data Availability

The data that support the findings of this study are available from the corresponding author upon reasonable request.
